# Clinical characteristics of chronic rhinitis following stroke

**DOI:** 10.3389/fneur.2023.1081390

**Published:** 2023-01-23

**Authors:** Jae Eun Choi, Yeong Wook Kim, Sungju Jee, Min Kyun Sohn

**Affiliations:** ^1^Department of Rehabilitation Medicine, Chungnam National University, Daejeon, Republic of Korea; ^2^Chungnam Regional Cardiocerebrovascular Center, Chungnam National University, Daejeon, Republic of Korea

**Keywords:** rhinitis, stroke, retrospective study, autonomic symptoms, subcortex

## Abstract

**Background:**

We previously observed that patients with stroke complained of rhinitis symptoms that developed following the occurrence of stroke.

**Objectives:**

To investigate the relationship between chronic rhinitis (CR) and stroke.

**Methods:**

This retrospective study analyzed the medical records and questionnaires of patients with stroke who visited our outpatient clinic from June to December 2020. Stroke lesions were mainly classified as supratentorial, infratentorial, and supra/infratentorial lesions. Supratentorial lesions were further divided into cortex, subcortex, and mixed. Participants were screened for CR and were subsequently divided into the CR and non-CR groups. The Sino-Nasal Outcome Test questionnaire and a questionnaire on autonomic nervous system symptoms were administered to all patients.

**Results:**

Clinically evaluated indicators were not significantly different between the two groups. The number of patients with lesions in both the cortex and subcortex was significantly higher in the CR group than in the non-CR group. The risk of CR was higher in male patients with stroke than their female counterparts; additionally, the risk of CR was higher in patients with stroke who had both cortical and subcortical lesions, as well as autonomic dysfunction.

**Conclusions:**

Individuals with subcortical stroke damage had a greater probability of developing CR. The risk was increased in men, as compared with that in women, when autonomic symptoms were present.

## 1. Introduction

Stroke is the third leading cause of death and disability and the second leading cause of death worldwide (1, 2). It is also the second leading cause of disability-adjusted life years in developing countries and the third leading cause in developed countries (after ischemic heart disease and back/neck pain) (3, 4).

We previously observed that, at outpatient clinic visits after discharge, patients with stroke, who had no history of allergic diseases, complained of rhinitis symptoms that developed following the occurrence of stroke. Most patients complained of discomfort during meals because of rhinorrhea. These symptoms could be defined as non-allergic rhinitis, a type of chronic rhinitis (CR) that causes rhinorrhea, nasal obstruction, sneezing, and/or itchy nose without any clinical evidence of infection or allergic diseases (5). Non-allergic rhinitis is affected by the sympathetic and parasympathetic nervous system of the nasal cavity, and rhinitis symptoms are thought to develop due to autonomic dysfunction that occurs after stroke (6).

It is well known that cerebrovascular diseases, particularly ischemic stroke, can either acutely or chronically alter the function of the autonomic nervous system (7–9). Autonomic dysfunction can also cause sino-nasal symptoms. While no studies on nasal symptoms have been conducted, among autonomic symptoms that occur after stroke, there have been cases of patients reporting symptoms of rhinorrhea following a stroke. Chen et al. reported a case of a 57-year-old Taiwanese male patient with ischemic stroke who had lesions in the right caudate nucleus and developed contralateral rhinorrhea. The patient complained of rhinorrhea on the left side after mastication or gustation at 2 months after the onset of cerebral infarction (10). Another case was a 74-year-old female patient with ischemic stroke who had lesions in the right lateral medulla and inferior cerebellum. She had episodes of clear secretions from her nose about 1 month after the onset of ischemic stroke. The otolaryngological evaluation did not reveal a clear cause of rhinorrhea (11).

Rhinitis symptoms that newly occur after stroke can be confusing and difficult for patients to manage. Non-allergic rhinitis has clinical symptoms that are similar to those of allergic rhinitis and equally or further worsen the quality of life (QOL). Early diagnosis and appropriate management of rhinitis can help improve the QOL of patients with stroke (12, 13). Therefore, the present study aimed to investigate the characteristics of CR in patients with stroke and determine whether the occurrence of CR is related to a specific lesion site. This study also subjectively evaluated autonomic dysfunction after stroke to confirm its relationship with the occurrence of CR.

## 2. Materials and methods

### 2.1. Participants

This survey was conducted by reviewing the medical records and questionnaires of patients who visited the outpatient clinic in the Department of Physical Medicine and Rehabilitation at the Chungnam National University Hospital (Daejeon, Korea) from June 1, 2020, to December 31, 2020. This study was approved by the hospital's Institutional Review Board (approval number: IRB 2020-01-059-006).

### 2.2. Data collection

The electronic medical records of the participants were reviewed, and necessary clinical data were collected. Demographic data such as age, sex, and smoking history were obtained, and scores for the Korean version of the National Institutes of Health Stroke Scale (K-NIHSS), Korean version of the modified Barthel Index (K-MBI), modified Rankin Scale (MRS), Functional Ambulatory Category (FAC), and Korean Mini-Mental Status Examination (K-MMSE) were examined as clinical data at the time of stroke onset. Patients were considered to have a smoking history if they had smoked at least once (14).

Additionally, stroke lesions were recorded based on patients' brain magnetic resonance imaging or computed tomography results. Lesions were divided according to lesion site into supratentorial, infratentorial, and supra/infratentorial (lesions in both supra- and infratentorial regions) regions. Supratentorial lesions were further divided into Supra_Cortex, Supra_Subcortex, and Supra_mixed, which included both subcortical and cortical locations (15).

### 2.3. Questionnaire

Participants were judged to have CR if they displayed two out of four symptoms, including nasal obstruction, rhinorrhea, sneezing, and itchy nose/eyes, for at least 12 weeks for 1 h or more daily. The questionnaire was used to evaluate whether the symptoms of CR appeared after the occurrence of stroke. Only those participants who responded “yes” to this question were included in the CR group. Based on this, the participants were further divided into the CR and non-CR groups (16). The characteristics of rhinorrhea, among the symptoms of CR, were recorded for patients assigned to the CR group.

The Sino-Nasal Outcome Test (SNOT-22) questionnaire, consisting of 22 CR-related questions, was administered to all participants and was used to evaluate the severity of nasal symptoms and their effect on the QOL. The questionnaire is divided into two parts: 12 questions on physical symptoms (rhinologic symptoms, cough, ear fullness, and facial symptoms) and 10 questions on QOL (sleep, fatigue, and mood). All questions were scored from 0 to 5, with 0 indicating no problem and 5 indicating a very serious problem. The questionnaire has a total minimum score of 0 and a maximum score of 110 points (17, 18). Notably, a high SNOT-22 score indicates low QOL and severe symptoms (19).

A questionnaire evaluation of autonomic symptoms was also administered to all participants to compare changes in the patients' autonomic function before and after stroke onset. The questionnaire consisted of questions about symptoms arising from autonomic dysfunction reported by Ewing et al. (20, 21). The questions included symptoms that occur while the participant assumed a standing position (palpitation, blurred vision, gastrointestinal discomfort, dizziness, and sticky skin), symptoms related to perspiration (increased or decreased perspiration in certain areas and increased perspiration in meals), and gastrointestinal symptoms (diarrhea, fullness, and bowel control), and the participants selected from 0–2 points based on the degree of subjective changes experienced during the past month compared to their status before stroke onset. In terms of scoring, 0 was selected if there was no change in symptoms, 1 was selected if the change in symptoms caused some problems in the patient's life, and 2 was selected if the change in symptoms always caused problems (20, 21).

The following rhinorrhea symptoms were confirmed in the CR group: the side of nasal discharge according to the side of stroke lesion, color of nasal discharge, viscosity of nasal discharge, time of nasal discharge, possibility of discharge occurring in a specific season or place, and improvement of symptoms (Table 1).

**Table 1:** Clinical characteristics of rhinorrhea in the chronic rhinitis group.

**Characteristic**		
Color	Clear	31
	Yellow	0
	Green	0
	Others	0
Viscosity	Thin	24
	Moderate	7
	Thick	0
Time	During meal	25
	Anytime	5
	Etc.	1
Correlation with season or place	Yes	9
	No	22
Side	Rt.	5
	Lt.	13
	Both	13
Side compared with Stroke lesion	Ipsilateral	8
	Contralateral	9
	Both	13
	Undecided	1

### 2.4. Statistical analysis

An independent *t*-test or the Mann-Whitney U test was used to compare age and clinical evaluation data, including K-NIHSS, K-MBI, MMSE, FAC, MRS, and SNOT-22 scores, between the two groups. The chi-squared test or Fisher's exact test was used to determine the correlation between categorical data in the CR and non-CR groups. Logistic regression analysis was conducted to identify risk factors for CR. *P* < 0.05 were considered statistically significant, and all statistical analyses were performed using IBM SPSS Statistics for Windows version 26.0 (IBM Corp., Armonk, N.Y., USA).

## 3. Results

### 3.1. Study population

**Figure 1:**
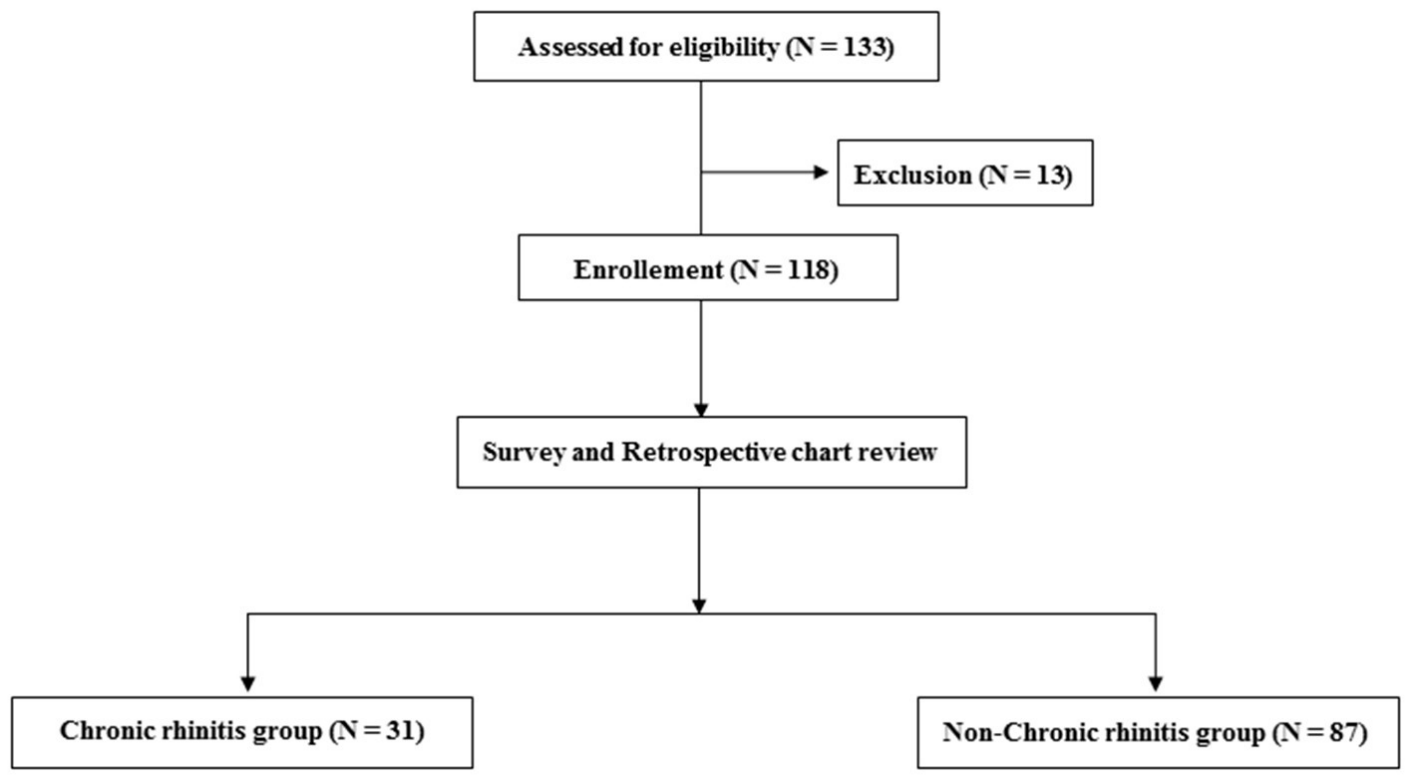
Flow chart depicting the number of included and excluded participants and the two groups of the included participants.

A total of 131 patients were assessed for eligibility in this study; however, 13 were excluded due to a history of allergic diseases before stroke onset. Among 118 participants who were enrolled, 31 and 87 participants were assigned to the CR and non-CR groups, respectively (Figure 1).

### 3.2. Demographic data and clinically evaluated indicators

Of the 31 participants, 27 (87.1%) in the CR group were men, which was significantly higher than that of the non-CR group, in which 54 of the 87 participants (62.1%) were men (*P* = 0.01). There was no significant difference in the mean age between the two groups (*P* = 0.52). The SNOT-22 score in the CR group was significantly higher (*P* = 0.04) than that in the non-CR group. Participants with smoking history were 14 (45.2%) in the CF group and 31 (35.6%) in the non-CR group, showing a similar distribution (*P* = 0.348). The K-NIHSS, K-MBI, and MMSE scores were not significantly different between the two groups (*P* = 0.487, *P* = 0.63, and *P* = 0.551, respectively). For the FAC and MRS scores, the participants were divided into two groups according to their ability to walk independently or not, but walking was shown to be significantly related to CR (*P*=0.934 and *P* = 0.625, respectively).

### 3.3. Clinical characteristics related to stroke

Participants with ischemic and hemorrhagic stroke were 22 (71.0%) and 9 (19.0%) participants, respectively, in the CR group, and 67 (77.0%) and 20 (23.0%) participants in the non-CR group, respectively, displaying no significant difference (*P* = 0.502). In terms of whether the stroke was first-time or recurrent, 24 (77.4%) and 7 (22.6%) participants in the CR group had a first-time stroke and recurrent stroke, respectively, whereas 79 (90.8%) and 8 (9.2%) participants in the non-CR group had a first-time stroke and recurrent stroke, respectively. There was no significant difference in the distribution between the two groups (*P* = 0.055).

When the stroke lesions were divided into right, left, and bilateral, there was no significant difference in the distribution between the chronic and non-CR groups (*P* = 0.718). Supra_Subcortex and Supra_mixed accounted for 22 of the 31(70.9%) participants in the CR group, which was significantly higher than 39 of the 87 (44.8%) participants in the non-CR group (*P* = 0.025; Table 2). Furthermore, only 4 (12.9%) patients had cortical lesions in the CR group, which was a significantly lower distribution than that of the 22 (25.3%) patients in the non-CR group.

**Table 2:** Participants' demographics, smoking history, SNOT-22 score, and clinical characteristics related to stroke (*N* = 118).

		**Chronic rhinitis**	**Non-chronic rhinitis**	**P-value**
Participants		31 (26.3%)	87 (73.7%)	
**Demographics**				
Sex, (M:F)		27 (87.1%): 4 (12.9%)	54 (62.1%): 33 (37.9%)	0.01^*^
Age, years		63.00 ± 9.61	64.57 ± 12.32	0.52
Diabetes mellitus		9 (29.0%)	26 (29.9%)	0.929
Smoking history		14 (45.2%)	31 (35.6%)	0.348
SNOT-22		18.29 ± 14.43	9.59 ± 11.13	0.04^*^
**Stroke type**				
Ischemic		22 (71.0%)	67 (77.0%)	
Hemorrhagic		9 (29.0%)	20 (23.0%)	0.502
**First/recurrent stroke**				
First		24 (77.4%)	79 (90.8%)	
Recurrent		7 (22.6%)	8 (9.2%)	0.055
**Stroke lesion**				
Supra_Cortex		4 (12.9%)	22 (25.3%)	
Supra_Subcortex		13 (41.9%)	33 (37.9%)	
Supra_mixed		9 (29.0%)	6 (6.9%)	
Infratentorium		4 (12.9%)	20 (23.0%)	
(Supra) Infratentorium		1 (3.2%)	6 (6.9%)	0.025^*^
Right		16 (51.6%)	46 (52.9%)	
Left		11 (35.5%)	34 (39.1%)	
Both		4 (12.9%)	7 (8.0%)	0.718
**Initial clinical evaluation**				
K-NIHSS		5.00 ± 4.46	4.36 ± 4.00	0.487
K-MBI		19.93 ± 8.47	20.78 ± 7.96	0.63
K-MMSE		53.97 ± 25.92	57.05 ± 23.41	0.551
**FAC**				
	0,1,2	17 (65.4%)	55 (66.3%)	
	3,4,5	9 (34.6%)	28 (33.7%)	0.934
**MRS**				
	0,1,2,3	8 (32.0%)	31 (37.3%)	
	4,5,6	17 (68.0%)	52 (62.7%)	0.625
Autonomic symptoms		27 (87.1%)	54 (62.1%)	0.010^*^

Data are presented as n (%) or mean ± standard deviation.

SNOT-22, Sino-Nasal Outcome Test; SD, standard deviation; K-NIHSS, Korean version of the National Institutes of Health Stroke Scale; K-MBI, Korean version of the modified Barthel Index; K-MMSE, Korean Mini-Mental State Examination; FAC, Functional Ambulatory Category; MRS, modified Rankin Scale.

^*^P < 0.05 indicates a statistically significant difference.

### 3.4. Clinical characteristics of rhinorrhea

The characteristics of rhinorrhea in patients in the CR group are summarized in Table 1.

### 3.5. Risk factors for CR

A logistic regression analysis was performed to determine whether the site of stroke lesion, sex, and autonomic dysfunction were potential risk factors for CR. The Nagelkerke R^2^ was confirmed to be 0.315, and *P* = 0.853 was obtained in the Hosmer-Lemeshow goodness-of-fit test, suggesting that the model fitted the data. An overall predictive value of 78.0% was obtained. The risk of developing CR was 12.369 times higher in patients with stroke with lesions in both the cortex and subcortex than in patients with lesions in only the cortex (*P* = 0.003). Furthermore, the risk of developing CR was 4.527 times higher in men than in women (*P* = 0.017) and 6.173 times higher in participants with autonomic symptoms than in participants without autonomic symptoms (*P* = 0.005; Table 3).

**Table 3:** Risk factors for chronic rhinitis.

**Covariate**	**Stroke lesion**	**B**	**S.E**.	**Exp(B)**	**95% CI**	**P-value**
Stroke lesion	Supra_Cortex			1.0		
	Supra_Subcortex	0.860	0.673	2.364	0.631–8.848	0.201
	Supra_mixed	2.515	0.860	12.369	2.293–66.717	0.003^*^
	Infratentorium	−0.132	0.807	0.876	0.180–4.265	0.870
	Supra_Infratentorium	0.176	1.273	1.1934	0.098–14.473	0.890
Autonomic symptoms	No			1.0		
	Yes	1.904	0.684	6.713	1.757–25.652	0.005^*^
Sex	Female			1.0		
	Male	1.510	0.631	4.527	1.314–15.596	0.017^*^
Constant		−4.376	1.009	0.013		0.000^*^

^*^P < 0.05 indicates a statistically significant difference.

### 3.6. Autonomic dysfunction

The number of participants who reported subjective changes in autonomic symptoms in one of the following areas, including cardiovascular, sudomotor, and gastrointestinal, in the autonomic symptom questionnaires was 27 of the 31 participants (87.1%) in the CR group, which was significantly higher number than 54 participants (62.1%) in the non-CR group (*P* = 0.01; Table 2). Among the 31 participants in the CR group, 16 participants reported abnormal symptoms when standing, 19 participants reported abnormal perspiration, and 18 participants complained of gastrointestinal symptoms. Among the 54 participants in the non-CR group, 32 reported abnormal symptoms when standing, 21 participants reported abnormal perspiration, and 34 participants complained of gastrointestinal symptoms. In the CR group, the number of participants who complained of gastrointestinal symptoms was significantly higher when the stroke lesion was on the right side than when it was on the left side (*P* = 0.001). Of the 118 participants in this study, 81 (68.6%) reported subjective changes in autonomic function after stroke, and there was no correlation (three symptoms: *P* = 0.351, *P* = 0.3245, and *P* = 0.214, respectively; Table 4) between the occurrence of each of the three symptoms and the location of the stroke lesion (right, left, or both). Among the patients who complained of subjective changes in autonomic function after stroke, 24 patients (29.6%) were diabetic. In the patient group that said there was no change in autonomic nervous system symptoms, 11 patients (29.7%) were diagnosed with diabetes (P = 0.991). It was confirmed that there was no association between the presence of autonomic nervous system symptoms and the presence of diabetes.

**Table 4:** Autonomic function evaluation by brain lesion.

	**Cardiovascular symptoms**	**Sudomotor symptoms**	**Gastrointestinal symptoms**	**Any changes**
**Chronic rhinitis**				
Right	9 (56.3%)	12 (63.2%)	14 (77.8%)	15 (55.6%)
Left	6 (37.5%)	5 (26.3%)	4 (22.2%)	9 (33.3%)
Both	1 (6.3%)	2 (10.5%)	0 (0.0%)	3 (11.1%)
*P*-value	0.621	0.277	0.001^*^	0.427
**Non-chronic rhinitis**				
Right	20 (62.5%)	13 (61.9%)	15 (44.1%)	29 (53.7%)
Left	10 (31.3%)	7 (33.3%)	17 (50.0%)	21 (38.9%)
Both	2 (6.3%)	1 (4.8%)	2 (5.9%)	4 (7.4%)
P-value	0.368	0.686	0.246	1.000
**Brain lesion**				
Right	29 (60.4%)	25 (62.5%)	29 (55.8%)	44 (54.3%)
Left	16 (33.3%)	12 (30.0%)	21 (40.4%)	30 (37.0%)
Both	3 (6.3%)	3 (7.5%)	2 (3.8%)	7 (8.6%)
P-value	0.351	0.324	0.214	0.800

^*^P < 0.05 indicates a statistically significant difference.

## 4. Discussion

Symptoms of autonomic dysfunction after stroke have been confirmed in several studies; nonetheless, studies reporting nasal symptoms, excluding case reports, have not been reported. The purpose of this study was to determine the occurrence of CR symptoms such as rhinorrhea and nasal obstruction in patients with stroke and to evaluate the risk that stroke lesions and sex pose on the morbidity of CR. Logistic regression analysis showed that the location of the lesion (Supra_Subcortex and Supra_mixed), presence of autonomic symptoms, and sex were risk factors for CR in patients with stroke. Age was not a risk factor for CR in patients with stroke.

Compared with patients with stroke who have lesions in the cortex, the risk of CR was 12.369 times higher in patients with stroke lesions in both the cortex and subcortex. According to Chen et al. (10), a patient with right caudate cerebral infarction developed contralateral rhinorrhea 2 months after stroke onset. They reported that damage to the caudate nucleus in patients with caudate stroke affected the superior salivatory nucleus, which induced reflex nasal secretion. In this study, patients with subcortex damage, including those with stroke lesions in both the cortex and subcortex (Supra_mixed), showed a higher risk of CR. Therefore, it can be inferred that the presence of lesions in the subcortex, including the basal ganglia, is related to the occurrence of CR; however, it is unknown whether rhinitis symptoms occur simply due to the large size of the lesion.

Several studies have suggested a relationship between specific stroke lesions and autonomic symptoms. Purwata et al. reported that the left hemisphere regulates parasympathetic modulation. Further, the left hemisphere stroke lesions were positively correlated with erectile dysfunction (22). In animal experimental studies, it has been reported that the head of the caudate nucleus showed a dual effect on secretory reflex, and it is thought that the inhibitory effect occurs through the action of acetylcholine on the dorsal part of the head of the nucleus and stimulatory effect through the action of adrenaline (23, 24). Among the participants with CR, the side of rhinorrhea was ipsilateral to the stroke lesion in eight participants, contralateral to the lesion in nine participants, present on both sides in thirteen participants, and not specific in one participant, suggesting that most participants experienced rhinorrhea on both sides. This study did not identify any correlation between the side of CR symptoms and side of the lesion.

The autonomic nervous system maintains physiological homeostasis and is composed of the sympathetic nervous system and parasympathetic nervous system. Both have a central nervous system and peripheral nervous system components. In addition to stroke, the causes of autonomic dysfunction are diverse, including primary causes such as Parkinson's disease, multiple system atrophy, and Lewy body dementia, and secondary causes such as diabetes, amyloidosis, and immune-mediated diseases (25). We found that more than half of patients with stroke complained of autonomic dysfunction after stroke onset, with 68.6% of all patients complaining of subjective autonomic dysfunction. As expected, the prevalence of CR was 6.173 times higher in patients with autonomic dysfunction than in patients without autonomic dysfunction. The symptoms of CR reported in this study can be viewed as complications caused by autonomic dysfunction after stroke (6).

Meyer et al. confirmed that norepinephrine was increased in patients with cerebral infarction compared to the control group and patients with a transient ischemic attack. This was not related to blood pressure and age (26). Studies have also reported that a right-sided ischemic stroke is more potent in increasing sympathetic function than a left-sided ischemic stroke (27, 28). In this study, changes in autonomic symptoms were subjectively evaluated, and its relationship with right and left lesions was analyzed (Table 4), but no significant relationship was found. According to Im et al., even patients who do not display symptoms of autonomic dysfunction may have autonomic nervous system failure in an objective examination. Although no statistical correlation was observed between the subjective autonomic dysfunction in patients with stroke and an objective examination of the nervous system, it is necessary to confirm symptoms through an objective examination in future studies (21).

Rhinorrhea indicates an imbalance between parasympathetic and sympathetic nerve activities, and it occurs under dominant parasympathetic hyperactivity (6). A decrease in sympathetic tone or an increase in parasympathetic tone leads to the dilation of venous sinusoids, thereby causing nasal obstruction. In the case of a stroke lesion, it can be considered that an abnormality has occurred in the pathways related to the superior salivatory nucleus of the parasympathetic nerve. As mentioned above, the sympathetic nerve tone increases after stroke; however, the extent to which the tone increases after stroke onset remains unknown.

In other words, the risk of CR was high in patients with cortex or subcortex lesions or subjectively assessed autonomic dysfunction. Since the symptoms of chronic rhinitis, such as runny nose, tears, and sneezing, are controlled by the autonomic nervous system, it is thought that nasal symptoms may be affected in patients with autonomic dysfunction as a complication after stroke. The importance of subcortical brain areas in autonomic function has been confirmed by several earlier investigations. Previously, the brainstem, amygdala, nucleus accumbens, and pallidum have been linked to the modulation and maintenance of SNS tone (29). Studies in rabbits and rats employing regional cutaneous vascular flow as a substitute for sympathetic activity showed that amygdala neuronal inhibition decreased cutaneous vasoconstriction, emphasizing the role of the amygdala in the sympathetic pathway (30). Subcortical brain regions have also been implicated in regulating PNS function, and the amygdala and palladium have both previously been implicated in key parasympathetic tones (29).

In our study, the results were similar, but the risk of CR increased in lesions that invaded the cortex and subcortex together, not lesions that invaded only the subcortex. This result suggests that autonomic dysfunction, which is the cause of CR, is affected by the interaction between the subcortex and the cortex. The relationship between stroke lesions and the development of CR has not yet been clearly elucidated. Further studies are needed to confirm clear evidence between CR and stroke lesions. Large-scale studies with increased sample sizes are needed for the results of males having a higher risk of CR.

Non-allergic rhinitis can be subdivided into senile rhinitis, gustatory rhinitis, occupational rhinitis, hormonal rhinitis, drug-induced rhinitis, and idiopathic rhinitis (16). The non-allergic rhinitis we refer to in this article falls under the category of idiopathic rhinitis. Senile rhinitis, gustatory rhinitis, and occupational rhinitis could be excluded, considering the patient's medical history and time of onset, but hormonal rhinitis and drug-induced rhinitis could not be clearly excluded. There is a limitation in clarifying further classification because it was not possible to investigate the use of drugs that can cause specific endocrine disease or rhinitis, such as NSAIDs like aspirin and ibuprofen, and beta-blockers (16).

Similarly, the SNOT-22 score was high among participants in the CR group in this study, and it can be concluded that the participants in the CR group had reduced QOL or discomfort due to CR. Patients with allergic symptoms before the occurrence of stroke were excluded to prevent the inclusion of allergic rhinitis during patient enrollment. However, the lack of objective tests, such as the skin prick test and serum allergen-specific IgE, can be a limitation in this process. CR was diagnosed solely based on the examination of medical history without an objective examination, such as the skin prick test. Although the SNOT-22 shows that there is a difference in QOL due to the development of CR in patients with stroke, further research is necessary because this index is insufficient to evaluate the overall QOL. Subjective evaluation was performed to determine the presence of autonomic dysfunction. In future studies, if objective tests such as sympathetic skin response or R-R interval variation are included, it is considered that they can be used as clear evidence of autonomic dysfunction (8). Additionally, further research on drugs (anticholinergics) that can affect autonomic dysfunction needs to be conducted.

In conclusion, we confirmed that the risk of CR was high in patients with stroke lesions in the subcortex. The risk was also higher in men than in women and when accompanied by autonomic symptoms. We also found that these rhinitis symptoms reduced the patients' QOL. Therefore, it is necessary to identify risk factors related to CR and to improve symptoms for the long-term management of the QOL of patients with stroke. Since the evaluation of patients with CR and autonomic nervous system symptoms is subjective, a prospective cohort study, which includes an objective diagnosis and severity evaluation of symptoms, is planned in the future. Finally, although rare, nasal symptoms can also occur in bell's palsy, which could be a limitation because they were not identified in the questionnaire.

## Data availability statement

The raw data supporting the conclusions of this article will be made available by the authors, without undue reservation.

## Ethics statement

This study was approved by the Hospital's Institutional Review Board (approval number: IRB 2020-01-059-006) and written informed consent was obtained from all patients included in this study. The patients/participants provided their written informed consent to participate in this study.

## Author contributions

MS contributed to the conception of the study. JC, YK, SJ, and MS performed clinical assessments. JC organized the database and performed statistical analysis. All authors contributed to the manuscript revision, read, and approved the submitted version.
